# Das Fehlen des Körpers in der psychotherapeutischen Ausbildung: Qualitative Analyse von Gruppendiskussionen mit Studierenden

**DOI:** 10.1007/s00729-021-00170-9

**Published:** 2021-05-05

**Authors:** Eva Wimmer, Birgitta Schiller, Manfred Reisinger, Isabella Wagner, Jutta Fiegl, Kathrin Mörtl

**Affiliations:** Institut für Qualitative Psychotherapieforschung, Institut für Psychosomatik, Sigmund Freud Privatuniversität Wien, Freudplatz 1, 1020 Wien, Österreich

**Keywords:** Körper und Psychotherapie, Ausbildungsforschung, Qualitative Forschung, Gruppendiskussion, Austausch mit anderen Professionen, Interprofessionalität und Psychotherapie, Fehlen des Körpers, Body and Psychotherapy, Psychotherapy training research, Qualitative research, Group discussion, Exchange with other professions, Interprofessional approaches in psychotherapy, The missing body

## Abstract

Im Rahmen eines Forschungsprojekts zum Thema „Der Körper in der Psychotherapie“ an der Ambulanz der SFU Wien wurden mit Studierenden Gruppendiskussionen geführt. Die hier vorgestellten Ergebnisse der qualitativen Analysen zeigen, dass in Bezug auf den Körper in der psychotherapeutischen Ausbildung eine Lücke wahrgenommen wird. Diese Lücke zeigt sich in drei Phasen der Wissensaneignung (Streben nach Sicherheit, Fluidität des Wissens und Umgehen mit der Unsicherheit). Unterschiedliche Formen von Lernen (Fachwissen, Selbsterfahrung, Austausch mit Kolleg*innen) ermöglichen den Umgang damit und die kontinuierliche Weiterentwicklung von Wissen zu Körperthemen während der Ausbildung. Dialog und Austausch mit anderen Professionen mit körperlichem Schwerpunkt bleiben weitgehend ein Wunsch der Teilnehmenden. Wir adressieren die Wissensaneignung und die hier von den Studierenden wahrgenommenen Lücken und formulieren Thesen, welche Implikationen diese Lücken besitzen.

## Abstract (en)

As part of a research project on “The body in psychotherapy” at the SFU Vienna outpatient clinic, group discussions were held with students. The results of the qualitative analyses that are presented here show that a gap is perceived in psychotherapeutic training regarding the body. This gap manifests itself in three phases of knowledge acquisition (striving for security, fluidity of knowledge and dealing with uncertainty). Different forms of learning (specialist knowledge, self-awareness, exchange with colleagues) make it possible to deal with the gap and to continuously develop knowledge on body topics during the training. Dialogue and exchange with other professions with a physical focus remain largely a wish of the participants. We address the acquisition of knowledge and the gaps perceived by the students and formulate our hypotheses about possibilities for implications based on the analysis.

## Einleitung – Die Studie im Kontext

Die hier vorgestellte Studie ist Teil einer Forschungsinitiative im Rahmen der Ambulanzforschung an der SFU Wien. Im Zentrum des Programms steht die Frage nach dem Körper in der Psychotherapie in unterschiedlichen Zusammenhängen. Vom Anamnesegespräch in der Ambulanz, in das über einen neugestalteten Anamnesebogen der Körper verstärkt einbezogen werden soll, über das Bewusstsein für Körper und Psyche in der Psychotherapie auf Ebene von Klient*innen und Therapeut*innen, bis hin zu Psychosomatik und der Etablierung interprofessioneller Zusammenarbeit reicht das Spektrum der geplanten und begonnenen Forschungsprojekte.

In dem vorliegenden Beitrag wird auf eines dieser Projekte Bezug genommen und die Rolle des Körpers in der psychotherapeutischen Ausbildung in den Fokus gestellt. Die Studie fußt auf einer Reihe an der Erwachsenenambulanz der SFU Wien durchgeführter Gruppendiskussionen (Przyborski und Riegler 2020) mit Studierenden der Psychotherapiewissenschaft an der SFU Wien zum Thema „Der Körper in der Psychotherapie“, wobei der Begriff des Körpers als weiter gefasst verstanden wird und nicht auf die medizinisch-psychosomatische Ebene beschränkt bleibt. Die Differenzierung zwischen Körper und Psyche gilt in der Psychotherapie spätestens seit Uexküll (z. B. 2001) als überholt (Tschuschke 2017); dennoch wird sie in vielen Fällen aus analytischen, konzeptionellen oder didaktischen Gründen beibehalten, wenn auch als wenig greifbares, kontextuell eingebettetes und subjektiv konstruiertes Konzept (von Schlippe und Streek 2006). Mit den hier präsentierten ersten Ergebnissen unserer Analysen gehen wir der Fragestellung nach, auf welchen Ebenen, in welcher Weise und in welchen Kontexten ein subjektiv definierter, in der Gruppensituation verhandelter „Körper“ bzw. „Leib“ von den Studierenden in der Ausbildung wahrgenommen wird.

Der physisch-materielle Körper ist Teil der psychotherapeutischen (PT) Ausbildung auf mehreren Ebenen: Psychosomatik, sexuelle Störungslehre, Psychiatrie, Neurologie etc. haben fachlich-medizinisch-distanzierte Bezüge zum Körper (Streeck und von Schlippe 2006). Wissen um den Körper wird in der PT-Ausbildung auf drei Ebenen vermittelt: a) die fachlich-theoretische Ebene, b) die Ebene der Selbsterfahrung und c) die Ebene von Austausch und Dialog mit anderen Professionen, Disziplinen und therapeutischen Schulen. In den Ebenen a) und c) steht die Körper(lichkeit) der Klient*innen im Zentrum, die Körper und Leibe der Therapeut*innen bleiben oft ausgeblendet. Selbsterfahrung, die den Körper einbezieht, ist selten und findet auf freiwilliger Basis statt (Schiller et al. 2019). Zusätzlich entwickelt jede therapeutische Fachrichtung die Zugänge zum Thema Körper ständig weiter (Geißler 2005; Teichmann-Wirth 2002).

Die individuellen Ausbildungsprozesse in der Aneignung von theoretischem und klinischem Wissen in der Profession der Psychotherapie wurden in einer früheren qualitativen Studie bereits gut dokumentiert (Schiller et al. 2017). Demnach sind die Arten der Wissensaneignung in der Psychotherapieausbildung abhängig von (1) der Ausbildungsphase (frühe Teile der Ausbildung finden noch ohne selbstständige praktische Arbeit statt), (2) dem speziellen Angebot (Theorieseminare, Selbsterfahrung in Gruppen- oder Einzelsetting, Supervision der Praxis), und dem individuellen Typus der Studierenden wie sie mit Lücken im Ausbildungsprozess umgehen: (A) Strukturiert-planungsorientiert, (B) Kommunikativ-beziehungsorientiert, (C) Erfahrungssuchend-handlungsorientiert. Diese Aneignung von Wissen in Kombination mit persönlicher Entwicklung ist eines der zentralen Kennzeichen der psychotherapeutischen Ausbildung insgesamt (Tilkidhzieva et al. 2019; Orlinsky und Ronnestad 2005) und konnte auch in der vorliegenden Untersuchung nachvollzogen werden.

Unsere Forschung zum Körper in der Psychotherapie steht auch im Kontext einer gesamtgesellschaftlichen Veränderung von Körperlichkeit(en) und Leiblichkeit(en). Durch die Verpflichtung zur physischen Distanzierung aufgrund der Maßnahmen zur Eindämmung der CoViD-19-Pandemie wurden Adaptierungen im Forschungsprozess notwendig, die das Fehlen des Körpers ins Zentrum rückten. Insgesamt hat sich seit März 2020 die Körperlichkeit im Lehren, Lernen und der psychotherapeutischen Praxis stark verändert. Physische Abwesenheit und die Neustrukturierung von bisher körperlich erlebten Situationen üben starken Einfluss auf das Erleben und Erfahren in der Lehre und Ausbildung aus (Dittler und Kreidl 2021; Kolb 2020; Kollmer 2020).

## Methoden – Durchführung der Gruppendiskussionen und Analyse des Materials

Im Jahr 2019 wurde im Rahmen der Ambulanzforschung zum Thema *„Der Körper in der Psychotherapie“* an der Erwachsenenambulanz der SFU mit der Durchführung von Gruppendiskussionen begonnen, an denen Psychotherapeut*innen in Ausbildung unter Supervision teilnahmen, die im Rahmen ihres Studiums an der Psychotherapeutischen Ambulanz der SFU mit Klient*innen arbeiteten. Mit der Entwicklung erster Thesen in der Analyse des Materials erweiterten wir den Stichprobenkreis und luden auch Studierende ein, die noch keine praktische Erfahrung in der Arbeit mit Klient*innen hatten, um im Sinne eines *theoretical sampling* (Strauss und Corbin 1996) die Aussagekraft der Thesen zu vertiefen und zu erweitern. Die Teilnehmenden an den Gruppendiskussionen waren also einerseits Studierende des Propädeutikums, andererseits Ausbildungskandidat*innen unterschiedlicher psychotherapeutischer Methoden.

An der SFU besteht die Kooperation mit neun psychotherapeutischen Fachspezifika, die selbstständig und autonom die fachspezifische Ausbildung der Studierenden ab dem 5. Semester Bakkalaureat (bis zum Abschluss des Magisterstudiums) durchführen. Ausgebildet wird in den Methoden Psychoanalyse (Psychoanalytisches Seminar Innsbruck), Integrative Gestalttherapie (Institut für Integrative Gestalttherapie Wien), Individualpsychologie (SFU Wien), Systemische Familientherapie (Österreichische Arbeitsgemeinschaft für Systemische Therapie und Systemische Studien), Verhaltenstherapie (SFU Wien), Psychodrama (SFU Wien), Personzentrierte Psychotherapie (Institut für Personzentrierte Studien Arbeitsgemeinschaft für Personzentrierte Psychotherapie, Gesprächsführung und Supervision), Existenzanalyse (Österreichische Gesellschaft für Logotherapie und Existenzanalyse), Transaktionsanalyse (Institut für Transaktionsanalytische Psychotherapie). Das hier erhobene Material ist also nicht nur institutionsspezifisch, sondern erstreckte sich auf acht Ausbildungsinstanzen mit unterschiedlichen und autonom unterrichtenden Lehrenden und Supervisor*innen. Die Spezifität der Ergebnisse bezogen auf den institutionellen Rahmen der SFU wird im Anschluss an die Ergebnisse noch kritisch diskutiert.

Insgesamt wurden bisher in einem Zeitraum von 15 Monaten 15 Gruppendiskussionen durchgeführt^1^; daran nahmen jeweils zwischen 4 und 7 Personen teil. Die ersten fünf fanden in der Ambulanz oder dem Hauptgebäude der SFU Wien statt. Die Einschränkungen infolge der CoViD-19-Pandemie machten die Verlagerung der Gruppen in ein online Setting notwendig. Eine möglichst große Ähnlichkeit in der Durchführung wurde angestrebt, auch wenn Online-Diskussionen nach anderen Maßgaben und Grundsätzen ablaufen als im Face-To-Face-Setting (Abrams und Gaier 2017; Schieck und Ulrich 2016). Inhaltlich konnte die Qualität der Diskussionen beibehalten werden.

Aus den Diskussionen ergaben sich ca. 17 h Material, die im Forschungsteam zunächst mit einer Themenanalyse (Froschauer und Lueger 2020) analysiert wurden. Mit dieser Methode werden thematische Konzepte im Kontext erfasst, Eigenschaften und Dimensionen der Themen aufgearbeitet und manifeste Gehalte des Gesagten auf einer abstrahierten, konzeptionellen Ebene verstehbar. Unterstützt wurde die Themenanalyse durch die Software MaxQDA. Einzelne, für die Fragestellung besonders relevante und interessierende Abschnitte wurden transkribiert und mit einer Systemanalyse (Lueger 2010) interpretiert, die die latenten, handlungsleitenden Strukturierungen eines Systems zugänglich macht. Mit dieser Methodentriangulation konnten die manifesten Gesprächsinhalte mit den latenten Strukturen und feldspezifischen Konstruktionsprinzipien zum Thema Köper(lichkeit)/Leib(lichkeit) in Beziehung gesetzt werden (Froschauer und Lueger 2020).

Die Fragen in den Gruppendiskussionen wurden bewusst offen gestellt (Przyborski und Riegler 2020), die erste Gesprächsanregung erfolgte zum Thema „Körper in der Psychotherapie“ allgemein und wurde im Verlauf der Diskussion mit Nachfragen zu anderen interessierenden Themen ergänzt. Diese bezogen sich auf die Ausbildung, die eigenen körperlichen Erfahrungen in der Arbeit mit Klient*innen und die Quellen von Wissen über den Körper. Dieser wenig gesteuerte Verlauf der Diskussionen ermöglichte die Analyse anhand der Relevanzsetzungen durch die Teilnehmenden ohne die Struktur und die Schwerpunktsetzungen vorzugeben – eine Notwendigkeit für interpretierende Analysen (Froschauer und Lueger 2020).

## Ergebnisse

Die folgenden ersten Ergebnisse der Analyse der Gruppendiskussionen werden unter der Prämisse präsentiert, dass es nicht *den Körper/den Leib* in der psychotherapeutischen Ausbildung gibt, sondern vielmehr ein Spektrum von Körperlichkeit/Leiblichkeit, das kontextuell und situativ abhängig ist. Die Studierenden setzen den Körper während der Diskussionen in jeweils unterschiedliche Kontexte und interpretieren ihn jeweils unterschiedlich, je nachdem in welcher Phase der Ausbildung sie sich befanden, welches Wissen über Körper(lichkeit) bereits vorhanden ist und welche individuellen Erfahrungen sie mit Körper/Leib bereits gemacht hatten.

### Aneignung von Wissen über den Körper – Die wahrgenommene Lücke

Im Verlauf des Studiums ändern sich für die Studierenden Zugänge zu Wissen und Nichtwissen, was auf Phasen des Wissenserwerbs beruht, die sich auch in den Analysen gezeigt haben. Dabei handelt es sich um Entwicklungen des Erwerbs und der Verarbeitung von Wissen von zunächst einer Suche nach Klarheit und Eindeutigkeit (Phase 1), die in eine Phase der Infragestellung und dem Gefühl der Flüchtigkeit bisherigen Wissens übergeht (Phase 2). Die dritte Phase ist gekennzeichnet von Vertrauen in die eigenen Fähigkeiten zum Wissenserwerb und der Sicherheit, auch Lücken im Wissen füllen bzw. aushalten zu können. Diese Phasen bauen aufeinander auf, sind jedoch nicht chronologisch zu verstehen, sondern als zyklischer Prozess, der während der gesamten Ausbildung und auch danach abläuft.

Diese Phasen des Wissenserwerbs zeigen sich in den Gruppendiskussionen als prozesshafte Auseinandersetzungen mit Ideen zum Körper in der psychotherapeutischen Ausbildung. In den ersten Phasen des Wissenserwerbs stehen Wissen über den physischen Körper und eher abstrakte Ideen zu Körperlichkeit in der Psychotherapie im Zentrum. Die fachlichen Themen des Lehrplans wurden in den Diskussionen gestreift, daneben ging es um konkrete Umsetzungen (z. B. „Sport gegen Depression“) und körperbezogene Interventionen. Die Quellen für diese Auseinandersetzungen sind eigene Erfahrungen und die unmittelbare Umwelt, weniger die bisher absolvierten Aus- und Fortbildungen. Das System Universität spielt hier die Rolle einer Plattform, auf der die Individuen ihre Erfahrungen im Austausch mit gelernten Inhalten und mit Kolleg*innen auf die Probe. Im Kontext der Gruppendiskussion wird der „Körper“ in Bezug auf die Psychotherapie als ein konkretes Phänomen gedeutet, das in den Dienst der Gesundheit gestellt werden kann und sollte. Ein stark positiv konnotierter Zugang zu körperlichen Interventionen („Bewegung ist gut“) steht im Zentrum und macht hier einen noch bestehenden dualistischen Zugang deutlich, der sich im Verlauf der theoretischen und praktischen psychotherapeutischen Ausbildung abschwächt. Auf der Ebene des inter- und intraprofessionellen Austauschs zu körperlichen Themen stellen die Teilnehmenden Lücken und individuelle Bedürfnisse nach Weiterbildung fest.

Der Austausch zwischen Therapieschulen ist an der SFU durch die Lehrveranstaltung „Methodenwerkstatt“^2^ institutionalisiert und findet so für die Studierenden des Fachspezifikums regelmäßig statt. Dies wird von den Teilnehmenden als große Bereicherung der Ausbildung und als Erweiterung des eigenen Horizonts angesehen – nicht nur, was körperbezogene Themen betrifft.

Dem fachlichen Austausch und dem Dialog zwischen Professionen hingegen werden im Studium bzw. in der Ausbildung nach Ansicht der Teilnehmenden zu wenig Raum gegeben.

Ein wichtiger Anspruch dieses interprofessionellen Austauschs besteht aus Sicht der Diskutierenden darin, die Klient*innen bestmöglich auch in Hinblick auf körperliche Erkrankungen, Symptome und Beeinträchtigungen unterstützen zu können. Im Idealfall – so die These – findet dies in einem fest etablierten Netzwerk von Professionist*innen statt, in dem die Psychotherapie eine zentrale Rolle einnimmt. Die von den Teilnehmenden beschriebene Vielfalt der möglichen Kooperationen^3^ zur Unterstützung von Patient*innen untermauert einerseits die These von der Lücke und dem Wunsch nach dem Füllen derselben; sie macht andererseits gleichzeitig zuversichtlich, dass der Umgang mit der Lücke und fehlende Expertise(n) in Bezug auf den Körper zum Wohle der Patient*innen ausgeglichen werden können. In der neuen Generation von Psychotherapeut*innen herrscht eine große Bereitschaft, sich interdisziplinär mit größtmöglichem Nutzen für die Klient*innen zu vernetzen – jedenfalls weisen unsere Daten in diese Richtung.

Das Fundament für interprofessionellen Austausch auf Patient*innenebene kann bereits in der Ausbildung gelegt werden. Nach dem Abschluss wäre es so einfacher, sich zu orientieren, wenn interprofessionelle Zusammenarbeit gewünscht wird. Schon während des Studiums wäre es auf der Basis unserer ersten Erkenntnisse empfehlenswert, wenn interprofessionelle Teams und Intervisionen zwischen Professionen stattfänden – zum Beispiel mit Medizinstudierenden aus der Fakultät für Medizin der SFU. Wechselseitige Überweisungen und Befundbesprechungen mit Hausärzt*innen oder Psychiater*innen der Klient*innen sind für die Diskutierenden selbstverständlich, allerdings abhängig von der Bereitschaft zur Kooperation seitens der (Fach‑)Ärzt*innen. Ein weiteres Thema in diesem Zusammenhang war auch die Frage, ob körperliche Untersuchungen unbedingt notwendig für eine Psychotherapie sein sollten bzw. sind und um welche es sich dabei handelt. Hier war ein klares Bekenntnis für körperliche Befunderhebungen und Untersuchungen zu erkennen. Dies zeigt eine große Aufgeschlossenheit der nächsten Therapeut*innengeneration zur Integration körperlicher Aspekte in die therapeutische Arbeit und auch der Anerkennung der Grenzen von Psychotherapie, wenn es um körperliche Prozesse geht.

### Das Fehlen des eigenen Körpers

Eine weitere Lücke, die in der Analyse der Gruppendiskussionen bemerkt wurde, zeigt sich weniger auf der manifesten Gesprächsebene als auf der Ebene dessen, was nicht gesagt wird bzw. was im Kontext der Diskussionen nicht gesagt werden konnte: Die Körper der Teilnehmenden selbst. Auf sie wurde nur bei konkreter Nachfrage Bezug genommen und auf spezifische Themen fokussiert. Es zeigt sich eine Externalisierung des Phänomens „Körper“, der als „das andere“ wahrgenommen wird, als etwas, das man *hat*, nicht *ist. *Der Körper ist da draußen, Klient*innen haben einen Körper, deren Erkrankungen werden thematisiert, therapeutische Konzepte besprochen, die den Körper fokussieren, doch die Körper und Leibe der Therapeut*innen selbst scheinen keine Rolle zu spielen.

Auf der Basis der oben dargestellten Wissensvermittlung zum Thema Körper in der Selbsterfahrung ist davon auszugehen, dass zumindest ein Teil der Teilnehmenden sich mit dem eigenen Körper auf unterschiedlichen Ebenen auseinandergesetzt hat. Doch warum kann dann in einer Diskussion nicht – oder nur sehr abstrakt darüber gesprochen werden? Die Teilnehmenden erwähnen ihre eigenen Körper in Bezug auf Körpersprache, Sitzhaltung, Frischluft oder Müdigkeit, wenn sie danach gefragt werden. In den tiefergehenden Diskussionen, die sich um Wünsche und Vorstellungen zum Körper in der Psychotherapie drehen, geht es um die Körper von Klient*innen.

Körperliche Aspekte der Psychotherapie und der Ausbildung wie die eigene (auch) körperliche Gesundheit/Krankheit, Bereiche der körperlichen Selbstwahrnehmung, die über Empfindungen wie Hunger, Durst oder ein eingeschlafenes Bein hinaus gehen, bleiben ebenso im Dunkeln wie Geschlecht und Sexualität oder Körperfunktionen der Teilnehmenden. Diese Themen bilden daher noch eine Lücke dieser Analysen. In den Interpretationen hat sich gezeigt, dass Tabuisierungen, Scham oder Konventionen eine Rolle spielen können, den eigenen Körper nicht zu thematisieren. Auch eine Internalisierung des „Fehlens“ des Körpers in der Ausbildung und damit die Unmöglichkeit von Körperlichkeit der Therapeut*innen wäre eine These, die zu prüfen wäre. Weitere Forschung in diesem Bereich ist unbedingt notwendig.

## Diskussion

Die ersten Analysen der Gruppendiskussionen mit Studierenden der Psychotherapiewissenschaft an der SFU Wien haben eine Fülle von Themen hervorgebracht, die für die Aufarbeitung der Rolle des Körpers in der Psychotherapie wichtig sind bzw. sein können. Der erste Schwerpunkt wurde auf das Thema „Der Körper in der psychotherapeutischen Ausbildung“ gelegt. In ihrer Ausbildung können die Studierenden auf eine Fülle von Wissen zum Körper aus unterschiedlichen Quellen zugreifen, das ihnen auf mehreren Ebenen (Fachwissen, Selbsterfahrung, Austausch) vermittelt wird. Die Ergebnisse der hier vorgestellten Analysen zeigen hier relevante Verbindungen zu den drei Idealtypen der in der Einleitung erwähnten Studie zu Studierenden der Psychotherapie (Schiller et al. 2017).

Deutlich erkennbar wurde in der Untersuchung, welche Bereiche der Ausbildung aus der Sicht der Befragten weniger Körper(lichkeit) repräsentieren als vielleicht gewünscht oder notwendig und wie sie mit diesem Fehlen des Körpers umgehen. Einerseits zeigte sich in den Diskussionen eine Intensive Auseinandersetzung mit Themen des „Fehlens“ von Körper/Leib in unterschiedlichen Kontexten, andererseits fand ein großer Teil der Diskussionen in einem Umfeld statt, das aufgrund der Maßnahmen zur Eindämmung der CoViD-19-Pandemie die Auseinandersetzung mit Köper(lichkeit) und Leib(lichkeit) in das Zentrum rückte – für die Diskutierenden wie auch für die Analysierenden.

Das „Fehlen“ des Körpers in der psychotherapeutischen Ausbildung ist weniger die faktische Auslassung körperlicher Aspekte, sondern eher die subjektive Wahrnehmung der Studierenden, dass in manchen – nicht näher benannten – Bereichen – der Körper und körperliche Aspekte „fehlen“. Auch das Auslassen von körperlichen Aspekten der Therapeut*innen selbst in der Diskussion könnte in diesem Kontext gesehen werden, wobei hier noch intensivere Bearbeitung notwendig sein wird.

Interessanter als die ausdrückliche Wahrnehmung einer spezifischen Lücke sind also einerseits der Umgang mit dem Fehlen und andererseits das Fehlen des Körpers auf Ebene der Diskutierenden selbst. In den Analysen hat sich diesbezüglich vor allem gezeigt, dass ein Fokus in der Wissensvermittlung auf einer interdisziplinären und interprofessionellen Wahrnehmung des Körpers von den Studierenden als sinnvoll erachtet wird. So kann der Anspruch eines ganzheitlichen Blickes auf den Menschen gewahrt bleiben. Die Lücke zum Thema Körper kann in der Ausbildung nicht geschlossen werden – wie bei so vielen Aspekten müssen die angehenden Therapeut*innen lernen, damit umzugehen.

Die Etablierung von fachlichem Austausch zwischen Disziplinen und Professionen kann jedoch helfen, die eigenen Lücken zu erkennen und zum Wohle der Klient*innen Unterstützung zu suchen, wenn es um konkrete körperliche Fragestellungen oder körperliche Ergänzungen zur Psychotherapie geht. Die Grundlage dafür könnte – nach unserer Ansicht auf der Basis der Analysen – bereits während des Studiums bzw. der Ausbildung gelegt werden, indem Netzwerke aufgebaut und interdisziplinäre Arbeit von Anfang an gefördert werden. In der heterogenen Zusammensetzung der Gruppendiskussionen liegt daher das Potenzial, die Aushandlungsprozesse zum Thema Körper in unterschiedlichen Kontexten analysieren zu können und die Themen unabhängig von der Fachrichtung oder dem Fortschritt der Studierenden als relevant zu erkennen.

Die SFU bietet hier durch die Vielfalt von psychotherapeutischen Methoden und Lehre durch unterschiedliche fachspezifische Vereine sowie der recht neu etablierten Medizinischen Fakultät eine große Chance dieser Vernetzung. Trotz der Vielfalt unterschiedlicher fachspezifischer Richtungen in unserer Stichprobe, bleibt es eine Studie, die bisher nur die Rekrutierung von SFU Studierenden vornahm. Obgleich wir davon ausgehen, dass die Lehre durch unterschiedliche autonome Fachspezifika hier einer SFU-Spezifität der Ergebnisse entgegenwirkt, müssen für zukünftige Rekrutierungen auch Ausbildungskandidat*innen ohne SFU Bezug einbezogen werden. Vorerst gehen wir davon aus, dass die hier präsentierten Themen auch in anderen Ausbildungsgruppen relevant sein könnten. Eine Aufschlüsselung der Themen nach Ausbildungsstand, Fachrichtung und Form der Ausbildung könnte ein Anschlussprojekt aus der eher quantitativ orientierten Forschung sein.

## Footnotes

1 Die Erhebungen laufen weiter.

2 In dieser Lehrveranstaltung werden Fälle aus der Sicht unterschiedlicher Therapiemethoden gemeinsam mit Supervisor*innen aus unterschiedlichen Fachrichtungen besprochen.

3 Die von den Teilnehmenden genannten möglichen Kooperationen sind: Medizin, Ernährungsberatung, Sportwissenschaft, Atemtherapeutische Ansätze, Sexualtherapie/-pädagogik, Entspannungs‑/Achtsamkeits‑/Meditationstraining.
